# The Effect of Inorganic Nitrogen Fertilizers on the Quality of Forage Composed of Various Species of Legumes in the Northern Part of a Temperate Climate Zone

**DOI:** 10.3390/plants12213676

**Published:** 2023-10-25

**Authors:** Gintarė Šidlauskaitė, Žydrė Kadžiulienė

**Affiliations:** Institute of Agriculture, Lithuanian Research Centre for Agriculture and Forestry, Instituto al. 1, 58344 Akademija, Lithuania; zydre.kadziuliene@lammc.lt

**Keywords:** climate change, dry matter yield, forage nutritive value, grasses, legumes, nitrogen application

## Abstract

This study focuses on the effect of inorganic nitrogen fertilizers on the quality of perennial grasses. Both grasses and legumes are important in swards, and each type of grass has different biological and ecological properties. Legumes in multi-species swards, especially in their early ages, benefit other Poaceae grasses by improving their growth. When evaluating individual cuts over a three-year period, it was determined that the quality indicators of the forage were significantly influenced by the year of use, N fertilizer application, and the different species compositions of the swards. In many cases, N fertilizers significantly reduced the CP content while tending to increase MADF and NDF. Monoculture grass swards had the highest WSC content; in most cases, N fertilizers increased the WSC content in the forage. DMD was the lowest in the first year of use, specifically in the first cut. Our three-year experiment, which investigated twelve swards with different species compositions, demonstrated that legume grasses improved the quality indicators of forage and contributed to maintaining a more stable overall forage yield over the years. As the climate continues to become warmer, there is a growing need to study a wide range of plant species and different varieties suitable for local growth conditions.

## 1. Introduction

Global climate changes directly or indirectly affect ecosystems in which inevitable processes take place as a result of qualitative and quantitative changes in vegetation, microorganisms, and soil [1]. Climate warming can change both soil temperature and the soil’s water content, with the potential to affect soil N availability [2]. This temperature increase is responsible for abiotic stresses that drastically affect yield productivity in various regions [3,4], with nonlinear effects on potential nutritional quality [5]. Such stresses can interfere with germination, vegetative growth, dry matter partitioning, and quality [6]. Climate fluctuations, rising temperatures, changing precipitation, and other extreme climate events contribute to changes in plant diversity [7]. A changing climate creates the need to research newer plant varieties [8] that are more resistant to drought or prolonged flooding [9].

In Europe, permanent grasslands cover 34% of the total agricultural area [10] and grow grass or herbaceous fodder, forage, or energy purpose crops [11]. Globally, farmers have managed to increase the biomass productivity of grasslands to support livestock production, and they are either directly grazed or cut for fodder, typically as hay or silage, or a combination of all three. Management activities are primarily used to change the status of soil properties, thereby creating optimal conditions for plant growth. Recent cases reported by Chen et al. (2019) [12], based on plant mixtures and corresponding monocultures analyzed with 1001 paired observations from 121 publications, support the hypothesis that both SOC content and stock are on average 5 and 8% higher in species mixtures than in monocultures. This leads to a higher accumulated annual yield. Furthermore, perennial herbaceous plants play a significant role in grassland ecosystem services in preserving their biodiversity, reducing the need for nitrogen during fertilization [13] and the negative effect on the environment.

One of the tools to improve yield and yield quality is the use of legume grasses as mixtures. Due to the ability of legumes to fix biological nitrogen, multi-species swards with two or three functional groups [14] can maintain better growth properties than monoculture swards. Nitrogen is an important element for the cultivation of swards globally [15], and it restricts the area of cultivation, especially due to the high price of synthetic fertilizers.

Grass–legume mixtures are not as easy to manage as monoculture swards because grasses and legumes compete for light, water, and nutrients [16]. However, several studies have shown the potential advantages of legume–grass mixtures in achieving higher or more stable yields, even during unfavorable weather conditions [8,13], and their highly developed and strong root systems make legumes more drought- and frost-resistant than grasses.

Forage nutritive value is directly related to the content and availability of essential nutrients for the consumption of animals [16]. This process is defined as the potency of an animal to consume, assimilate, and digest the essential nutrients in feed. Various factors affect the nutritive value of forage; the productivity and quality of swards can change over time, and they depend on the age of the grasses, climatic conditions, direction of the farm, intensity of use, and other factors. Forage quality is generally measured in terms of crude protein (CP), neutral detergent fiber (NDF, for intake), digestible dry matter, or acid detergent fiber (ADF, for digestibility) [17]. Similar grasses can have different nutritive values in different years, even when each is planted in the same field [18]. In general, all forages are highly succulent during early growth, which markedly enhances their palatability. In addition, their high protein content in relation to a low fiber content at this stage makes them highly nutritious as livestock forage [19]. According to other researchers, plant functional traits can also impact the forage nutritive quality [20].

Grass–legume mixtures are recognized as having a higher crude protein content [21], as well as lower NDF fiber concentrations, than monoculture swards [18]. Lignin is a noncarbohydrate substance and is the main factor that influences the digestibility of the contents of the plant cell wall [22]. A low level of lignin in forages is desirable. Thus, leguminous forages have a better nutritive profile than grasses [23], with a balanced amino acid profile and a good proportion of vitamins and minerals. An example of this is the study carried out by McDonald (2021) [16] in which the average, over 2016 and 2017, of the relative feed value (RFV) was the highest in low-lignin alfalfa and the lowest in grass monocultures.

NDF values increase as the forage matures, and it is important to harvest grass vegetation at an early stage; forage with higher NDF values is less suitable for consumption by animals [24]. More than 60% of NDF content denotes a low quality of forage, as it interferes with the digestion and assimilation of nutrients by animals; however, a value below 40% indicates good quality [25]. Moreover, as ADF increases, digestibility and nutrient availability also decrease; hay with ADF levels above 45% has little nutritive value; therefore, a value lower than 31% indicates good quality. But the presence of high crude protein (CP) in forages makes rumen microflora more active [26] and increases the efficiency of livestock to digest and assimilate nutrients.

The aim of this study was to determine the effects of forage diversity and the nutritive quality of swards in a three-year multi-species grassland experiment with and without nitrogen application, using a cutting regime over the three-year period.

## 2. Results

### 2.1. Crude Protein

There were significant differences in the chemical compositions of the yields between the years; therefore, all the data are presented. Fertilization did not have a significant effect on the first cut of 2019, and no interaction between factors was found in the third cut of 2021 (Table 1).

**Figure 1:**
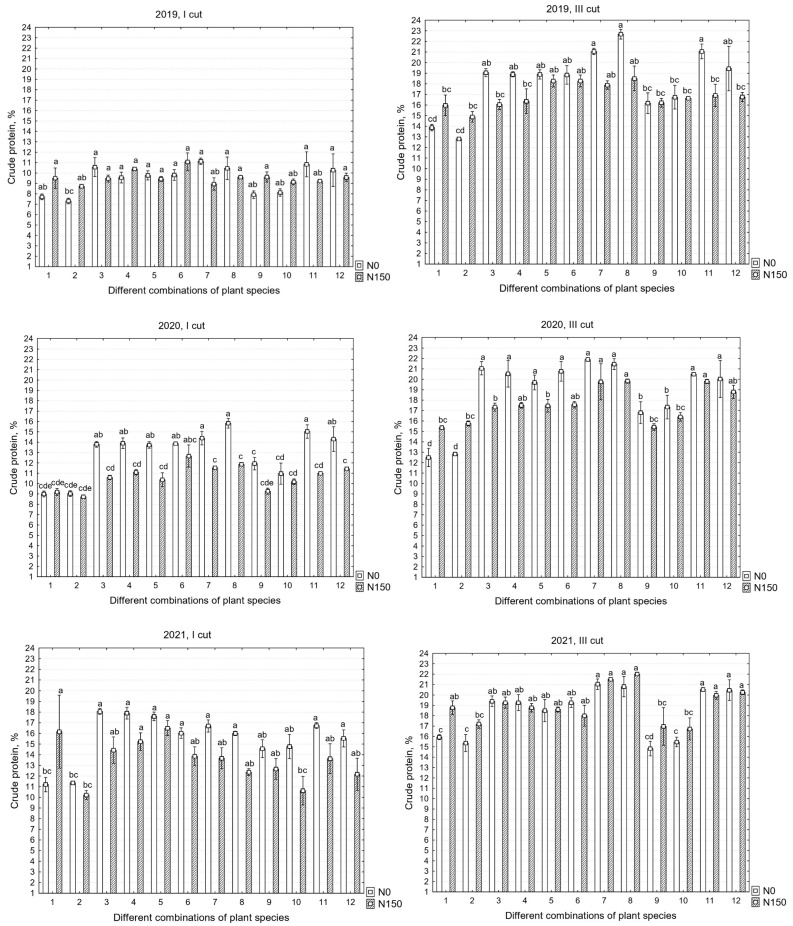
Crude protein in the first, second, and third years of sward use in different cuts. Treatments: 1—G1, 2—G2, 3—L1 + L2/G1, 4—L1 + L2/G2, 5—L1 + L2/G1 + G2, 6—L1 + L2/G1 + G2 + G3 + G4, 7—L3 + L1/G1 + G2, 8—L3 + L1/G1 + G2 + G3 + G4, 9—L4 + L1/G1 + G2, 10—L4 + L1/G1 + G2 + G3 + G4, 11—L1 + L2 + L3 + L4/G1 + G2, 12—L1 + L2 + L3 + L4/G1 + G2 + G3 + G4. Grasses: G1—perennial ryegrass, G2—×*Festulolium*, G3—meadow fescue, G4—timothy. Legumes: L1—white clover, L2—red clover, L3—lucerne, L4—sainfoin. Level of N fertilizer per year: N150; different letters indicate significant differences between the treatments (*p* < 0.05).

The crude protein (CP) contents of the I and III cuts in 2019–2021 are shown in Figure 1, where CP was found to range from 7.7 to 22.6% in dry matter (DM). In all cuts of the first two years, there was an interaction between species treatments and fertilizer applications for CP, but this did not occur in the third year. Observations revealed that the accumulated content of CP in the first cut was lower than in the third cut; the average data over the three-year period indicate that the first cut accumulated between 9.4 and 15.5% of CP, whereas the third cut ranged from 16.9 to 19%. In all cases, the different species compositions of the grasses had a significant impact on the content of CP in the swards. Furthermore, unfertilized swards with a more diverse species composition exhibited a higher nutritional value. The data indicate that, in different years of sward use, there was a varying impact of the mineral nitrogen fertilizers on separate cuts. It was determined that only in the first cut of the first year of sward use was there no significant effect on the content of CP in the swards. However, in the subsequent years, the application of mineral nitrogen fertilizers substantially reduced the content of CP, decreasing on average by about 17.9% in 2020 and 13.3% in 2021. In the third cut, it reduced the content by 7.7% and 6.4% in 2019 and 2020, respectively, but, in 2021, the mineral nitrogen fertilizers increased the average CP content by 3.3%. In some cases, excessive nitrogen fertilization can result in a shift from a legume-dominated community to a grass-dominated one [27]. The data do not show this; however, upon analyzing the annual botanical composition of the swards, it was determined that the absence of mineral nitrogen fertilizers directly resulted in a higher biomass of legume grasses than in fertilized swards. In 2019, legume grasses on average constituted 54% of the total sward biomass; this changed to 71% in 2020 and to 64% in 2021. Meanwhile, with the use of mineral nitrogen fertilizers, the corresponding percentages were 19%, 34%, and 46%, respectively. Furthermore, it was observed that distinct species of legume grasses in the sward influenced not only overall sward productivity but also the content of CP. For instance, the sown sainfoin had the lowest presence from 27 to 58% in separate years, which correlated with a lower CP content, while lucerne went from 72 to 76% (N_0_). Aside from white clover, when comparing other supplementary legume grasses in the mixtures, lucerne dominated more than red clover. However, the interaction of all three legume grasses did not significantly increase productivity or the content of CP indicators.

### 2.2. Modified Acid Detergent Fiber

There were significant differences in the chemical compositions of the yields between the years; therefore, all the data are presented. In the first cut, in all years, the mineral nitrogen fertilizer significantly increased the MADF content, but, in the third cut, a significant increase in MADF was found only in the second year of sward use (Table 2).

**Figure 2:**
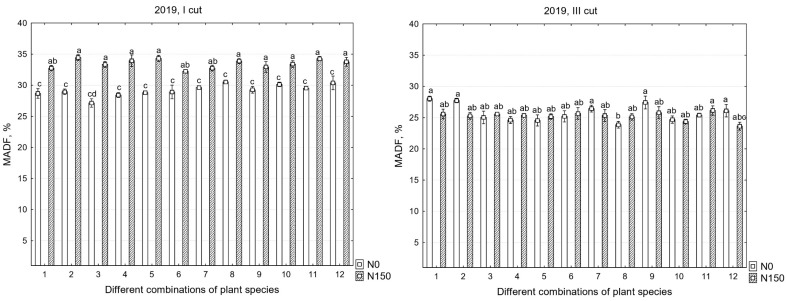

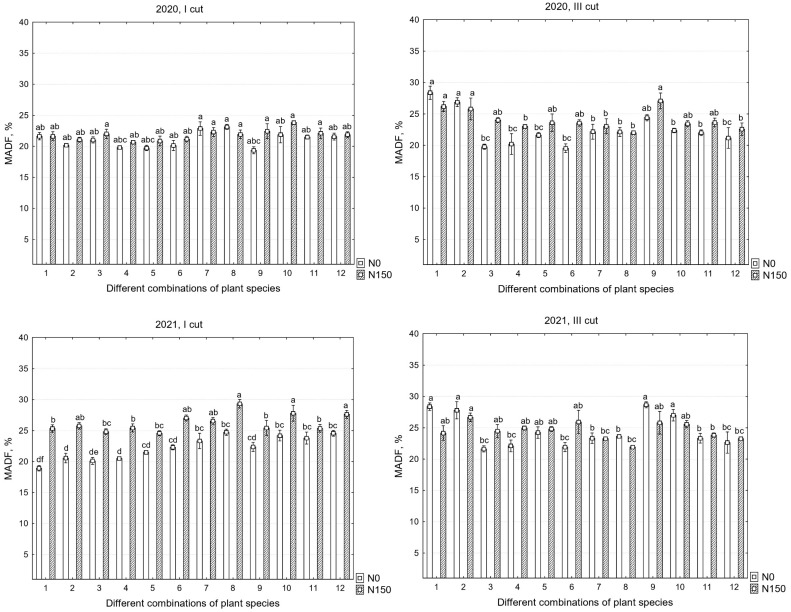
Modified acid detergent fiber in the first, second, and third years of sward use in different cuts. Treatments: 1—G1, 2—G2, 3—L1 + L2/G1, 4—L1 + L2/G2, 5—L1 + L2/G1 + G2, 6—L1 + L2/G1 + G2 + G3 + G4, 7—L3 + L1/G1 + G2, 8—L3 + L1/G1 + G2 + G3 + G4, 9—L4 + L1/G1 + G2, 10—L4 + L1/G1 + G2 + G3 + G4, 11—L1 + L2 + L3 + L4/G1 + G2, 12—L1 + L2 + L3 + L4/G1 + G2 + G3 + G4. Grasses: G1—perennial ryegrass, G2—×*Festulolium*, G3—meadow fescue, G4—timothy. Legumes: L1—white clover, L2—red clover, L3—lucerne, L4—sainfoin. Level of N fertilizer per year: N150; different letters indicate significant differences between the treatments (*p* < 0.05).

The modified acid detergent fiber (MADF) contents of the I and III cuts in 2019–2021 are shown in Figure 2. The data indicate that, in all experimental years, in the first cut, both the different species compositions of the grasses and fertilization had a significant effect on the MADF content; however, no interaction between these factors was identified. In the third cut, fertilization did not have a significant effect on MADF increase or decrease (except for in 2020), and the species composition of the grasses had a more pronounced influence on the changes in the MADF content in the swards. 

However, in all cases, the swards exhibited a high nutritional value regardless of the mode of utilization; a limit greater than 45% MADF content was not observed in any of the years, which indicates a reduction in nutrient availability and digestibility. The average MADF content in individual cuts ranged from 21.1 to 29.2% and from 21.8 to 33.5% when using and not using mineral nitrogen fertilizers, respectively. 

The three-year average data for the first cut (N_0_) show that the best digestibility was achieved in the monoculture swards with perennial ryegrass and ×*Festulolium* and in the mixtures of these grasses with red clover, with MADF contents ranging from 22.8 to 23.3%. Compared to the other swards, the only statistically significant difference in MADF content was found in the mixture of white clover and alfalfa with the other four species of grasses, where the MADF content was 26.1%; however, this is less than 30%, indicating that the nutritional value of the sward was excellent. In contrast, with N_150_, no statistically significant differences were found between the treatments and MADF contents, which ranged from 26.6 to 28.3%. Meanwhile, the annual averages of the third cut showed the opposite results; the monoculture swards and the mixture with sainfoin and two species of grasses had the same tendency to accumulate a higher MADF content, ranging from 25.3 to 28.3, and with both N_0_ and N_150_, compared to the other swards, this ranged from 22.1 to 24.7%.

### 2.3. Neutral Acid Detergent Fiber

There were significant differences in the chemical compositions of the yields between the years, except for in the third cut; therefore, all the data are presented. In the first cut, in all years, the mineral nitrogen fertilizer significantly increased the NDF content, as well as the MADF content; however, in the third cut, no significant increase in NDF was found in the third year of sward use (Table 3).

**Figure 3:**
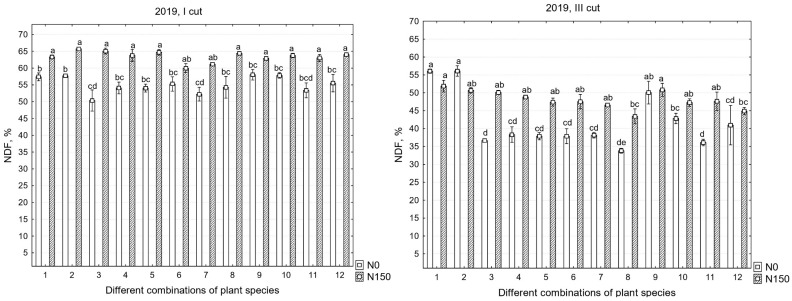

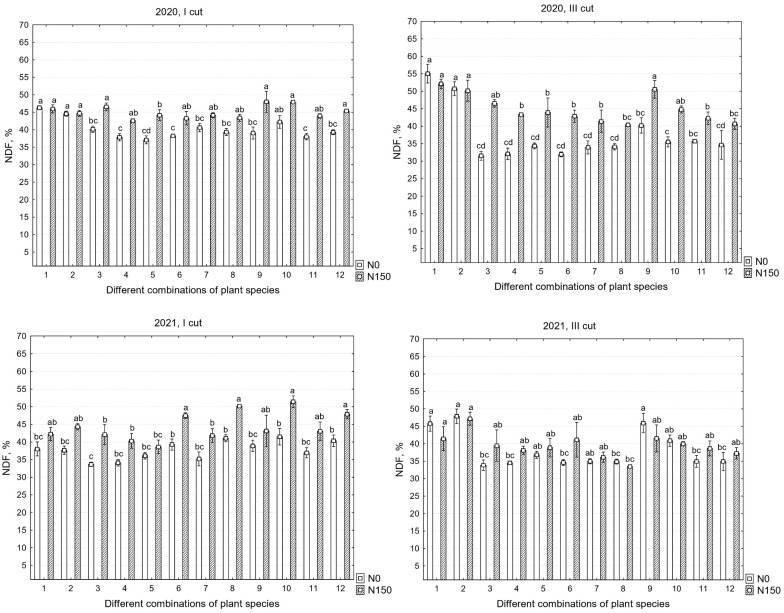
Neutral detergent fiber in the first, second, and third years of sward use in different cuts. Treatments: 1—G1, 2—G2, 3—L1 + L2/G1, 4—L1 + L2/G2, 5—L1 + L2/G1 + G2, 6—L1 + L2/G1 + G2 + G3 + G4, 7—L3 + L1/G1 + G2, 8—L3 + L1/G1 + G2 + G3 + G4, 9—L4 + L1/G1 + G2, 10—L4 + L1/G1 + G2 + G3 + G4, 11—L1 + L2 + L3 + L4/G1 + G2, 12—L1 + L2 + L3 + L4/G1 + G2 + G3 + G4. Grasses: G1—perennial ryegrass, G2—×*Festulolium*, G3—meadow fescue, G4—timothy. Legumes: L1—white clover, L2—red clover, L3—lucerne, L4—sainfoin. Level of N fertilizer per year: N150; different letters indicate significant differences between the treatments (*p* < 0.05).

Neutral detergent fiber (NDF) measures cell wall components, such as hemicelluloses, cellulose, and lignin, and it shows the intake potential. In general, increased NDF digestibility results in higher digestible energy and forage intakes. As shown in Figure 3, the highest NDF values were recorded in 2019, in the first cut with N_150_, where the average NDF content in the swards reached 63.5%. It was also observed that, in this cut, the different species compositions of the grasses did not have a significant impact on NDF levels. However, the mineral nitrogen fertilizers had a significant effect, increasing the NDF content by an average of 13.4%. When analyzing the third cut of 2019 with N_0_, a significant effect of the different species compositions was found. Two monoculture swards with perennial ryegrass and ×*festulolium* accumulated a significantly higher NDF content (NDF average of 56%) than the other grass–legume swards (NDF average of 39.3%). The lowest amount was in the sward with white clover, lucerne, perennial ryegrass, ×*festulolium*, meadow fescue, and timothy grass (NDF 33.8%). When using N_150_ in the swards, no significant differences were observed among the swards with various species compositions, but, as with the first cut, it significantly increased NDF content by 12.5% on average.

In 2020, the NDF content of the swards in separate cuts was more consistent compared to in 2019. The NDF content ranged from 40.2% to 38% in the first and third cuts with N_0_, respectively, and from 45% to 44.9% with N_150_; this indicates that the forage was more palatable than the first year of sward use.

Furthermore, there is a tendency in which mineral nitrogen fertilizers significantly increase the NDF content in swards in separate cuts, but, often, this is not dependent on the species composition of the sward. Although the data do not show statistically significant differences in sward productivity in separate cuts when using N_150_, this indicates that different species compositions did not have a significant impact on sward productivity, contrary to N_0_. A botanical analysis of the sward compositions also revealed that the use of mineral nitrogen fertilizers reduced the overall legume grass content. However, the grass monoculture swards accumulated statistically more NDF than the other grass–legume swards.

In the third year of sward use, in the first cut, as in previous years, it was observed that the nitrogen fertilizers significantly increased the NDF content, except for in the third cut. In the third cut, only the species diversity of the grasses had a significant effect on the NDF content changes in the swards.

### 2.4. Water-Soluble Carbohydrates

There were significant differences in the chemical compositions of the yields between the years; therefore, all data are presented. In the first cut, the mineral nitrogen fertilizer significantly decreased the WSC content in 2019 and increased it in 2020; however, in the third cut, a significant decrease in WSC was found only in the third year of sward use, which increased in 2019 and 2020 (Table 4).

**Figure 4:**
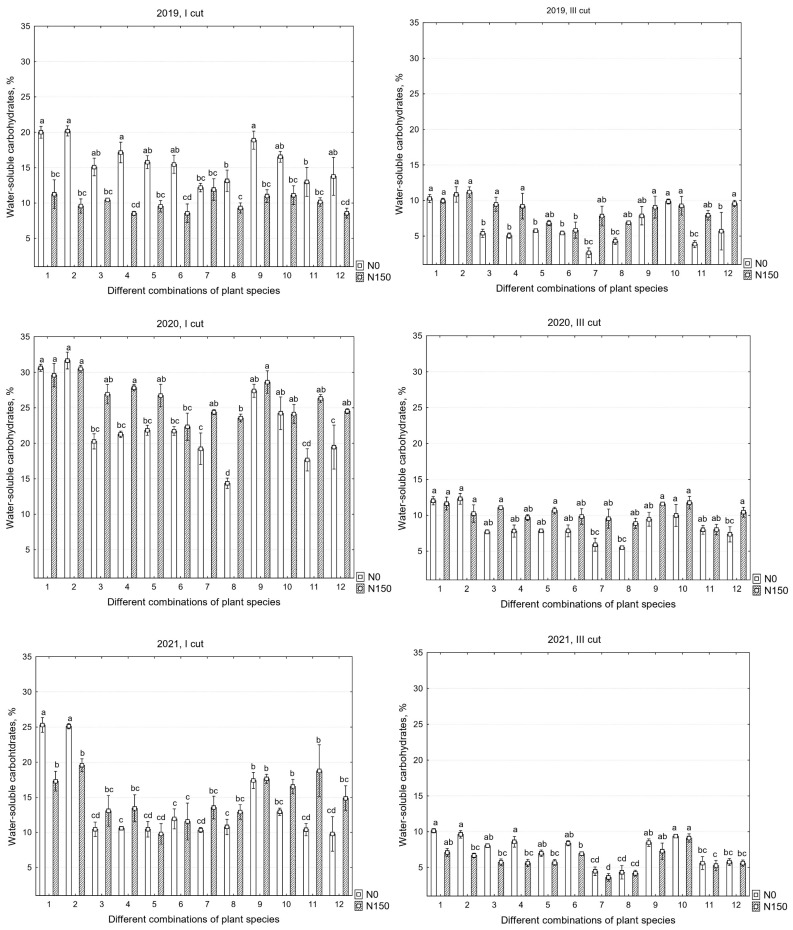
Water-soluble carbohydrates in the first, second, and third years of sward use in different cuts. Treatments: 1—G1, 2—G2, 3—L1 + L2/G1, 4—L1 + L2/G2, 5—L1 + L2/G1 + G2, 6—L1 + L2/G1 + G2 + G3 + G4, 7—L3 + L1/G1 + G2, 8—L3 + L1/G1 + G2 + G3 + G4, 9—L4 + L1/G1 + G2, 10—L4 + L1/G1 + G2 + G3 + G4, 11—L1 + L2 + L3 + L4/G1 + G2, 12—L1 + L2 + L3 + L4/G1 + G2 + G3 + G4. Grasses: G1—perennial ryegrass, G2—×*Festulolium*, G3—meadow fescue, G4—timothy. Legumes: L1—white clover, L2—red clover, L3—lucerne, L4—sainfoin. Level of N fertilizer per year: N150; different letters indicate significant differences between the treatments (*p* < 0.05).

The water-soluble carbohydrate (WSC) contents of the I and III cuts in 2019, 2020, and 2021 are shown in Figure 4. In all years of the study, statistically significant differences in WSC were observed in all cuts, both due to the different species compositions of the swards and the use of mineral nitrogen fertilizers, except for in the first cut in 2021. The data presented in Figure 4 indicate that there was a significant difference in the WSC content between the first and third cuts. In the first cut, when using N_150_, WSC was higher than in the third cut, as observed in 2019, 2020, and 2021; specifically, the average WSC contents ranged from 9.9% to 8.6%, 26.3% to 10.3%, and 14.9% to 6%, respectively. Meanwhile, with N_0_, the average WSC contents in the separate cuts were 15.9% to 6.4%, 22.5% to 8.5%, and 13.8% to 7.5%. In 2020, the first cut with N_150_ had the highest WSC content, which, in terms of forage quality and nutritive value, exceeded the recommended limit of not more than 20%; in individual swards, it ranged from 22.4% in mixtures to 30.5% in monocultures. Meanwhile, with N_0_, it ranged from 14.4% in mixtures to 31.7% in monocultures.

In the first two years of sward use, with N_0_, it was observed that, in the first cut, not only the monoculture swards of perennial ryegrass and ×*festulolium* accumulated the most carbohydrates but also the mixture of white clover and sainfoin with two species of grasses, with these swards accumulating average WSC contents of 19.7% and 29.9%, which significantly differed from the swards containing lucerne, where the average WSC was 13% and 17.7% in 2019 and 2020, respectively. It is also important to note that, as shown in Figure 1, these mixtures with lucerne accumulated significantly more crude protein than the monoculture swards.

### 2.5. Dry Matter Digestibility

There were significant differences in the chemical compositions of the yields between the years; therefore, all data are presented. In the first cut, the mineral nitrogen fertilizer significantly decreased the DMD content in 2019 and 2021; however, in the third cut, a significant increase in DMD was found only in the first year of sward use (Table 5).

**Figure 5:**
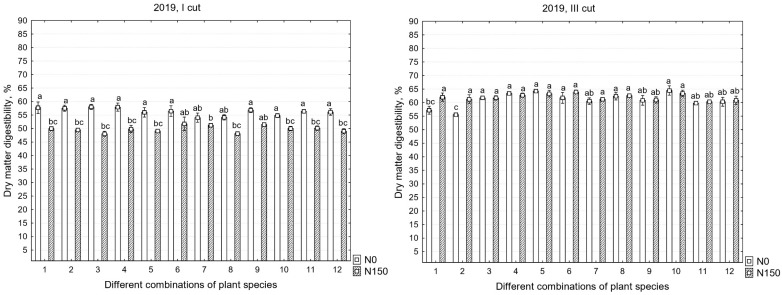

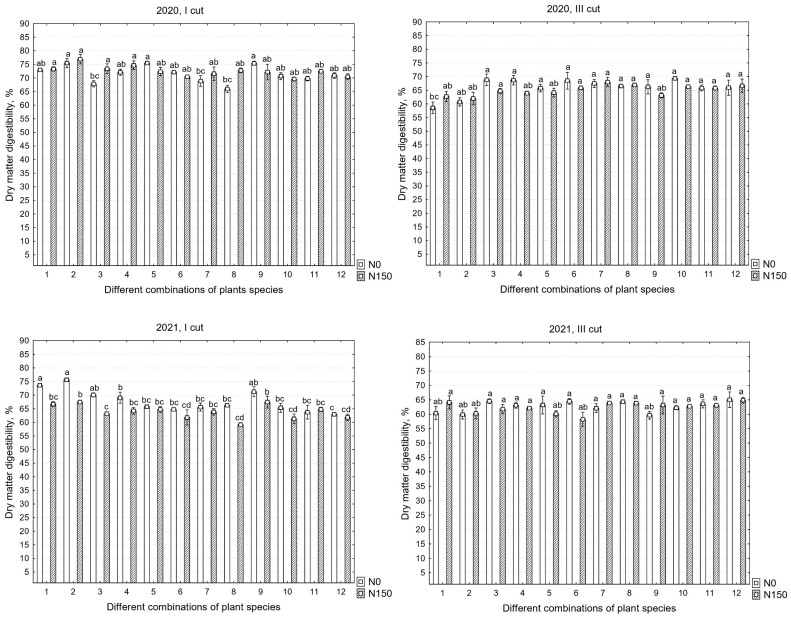
Dry matter digestibility in the first, second, and third years of sward use in different cuts. Treatments: 1—G1, 2—G2, 3—L1 + L2/G1, 4—L1 + L2/G2, 5—L1 + L2/G1 + G2, 6—L1 + L2/G1 + G2 + G3 + G4, 7—L3 + L1/G1 + G2, 8—L3 + L1/G1 + G2 + G3 + G4, 9—L4 + L1/G1 + G2, 10—L4 + L1/G1 + G2 + G3 + G4, 11—L1 + L2 + L3 + L4/G1 + G2, 12—L1 + L2 + L3 + L4/G1 + G2 + G3 + G4. Grasses: G1—perennial ryegrass, G2—×*Festulolium*, G3—meadow fescue, G4—timothy. Legumes: L1—white clover, L2—red clover, L3—lucerne, L4—sainfoin. Level of N fertilizer per year: N150; different letters indicate significant differences between the treatments (*p* < 0.05).

The data from the first cut show that the decrease in dry matter digestibility (DMD) content was significantly influenced only by fertilization in 2019; only by the different species compositions of the swards in 2020; and by both fertilization and the different species compositions of the swards in 2021, with an identified interaction between these two factors (Figure 5). The data show that, in the first year of sward use, in the first cut, the digestibility indices were the lowest compared to the other cuts, regardless of the method of sward use, averaging 56.3% with N_0_ and 49.9% with N_150_. When compared to the results of 2020 and 2021, the digestibility indices had already reached 71.5 and 72.5%, and 67.8 and 63.9%, respectively. When analyzing the third cut, it was found that the N fertilizers did not have a significant impact on the sward digestibility indices, except for in 2019.

## 3. Discussion

European grassland-based livestock production systems have undergone significant transformations in the past twenty years and will further adapt in response to societal and environmental challenges [28]. The hypothesis of this work was related to the fact that legumes present valuable opportunities for sustainable grassland-based animal production; they can address critical challenges by substituting the use of inorganic nitrogen fertilizers through symbiotic nitrogen fixation, increasing forage yield, enhancing the nutritive value of herbage, and improving the efficiency of converting herbage into animal protein. Legumes and grasses often compete for resources, such as light, water, and nutrients [29]. According to data from other researchers, the excessive use of mineral nitrogen can alter the composition and activity of soil microorganisms [30], which is essential for normal grass growth. Nitrogen fertilization might tip the competitive balance in favor of grasses, leading to reduced legume growth. Legume grasses are known for their high protein content and good protein quality. When added to a sward mixture, they can contribute to increasing the overall protein content. This is particularly beneficial for livestock feed production, as a high protein content in grass can enhance feed nutritional value. Previous studies have also reported [31] that legume grasses like white clover, red clover, and lucerne exhibit elevated levels of crude protein (CP) and minerals, including calcium; however, in comparison to perennial ryegrass (*Lolium perenne* L.), they have relatively lower concentrations of water-soluble carbohydrates (WSCs).

Water-soluble carbohydrates (WSCs) in forage refer to carbohydrates that dissolve in alkaline conditions, can easily dissolve in water, and are available for plant nutrition. WSC in forage can be an important parameter that indicates the amount of these carbohydrates in the feed composition [32]. Water-soluble carbohydrates in forage can be significant, especially in the livestock and dairy industries, as they can influence feed nutritive value, energy supply, and livestock productivity [27]. Plants with higher WSC levels can be beneficial for certain livestock species, but it is important to remember that excessive sugars can be detrimental to specific livestock breeds prone to conditions like diabetes. Schmidt et al., 2023, found that season and grass species composition have a greater effect on WSC concentrations in pasture than fertilizer treatment [32]. These results are similar to those reported by Thers and Eriksen, 2022, and Kagan et al., 2020 [33,34].

Different grass species have varying nutritional values. Lucerne, for example, is often a good source of protein [35], while perennial ryegrass or other grasses may have a different nutritional profile. Considering this, animals may require various nutrients, so dietary diversity can be important. Red clover and lucerne exhibit lower digestibility, with their net energy concentration being inferior to that of white clover at similar growth stages [31]. The difference is most significant for lucerne, with values of 5.54, 6.10, and 7.17 MJ kg DM^−1^ for lucerne, red clover, and white clover, respectively [36]. Our results are similar to those found by Elgersma and Søegaard, 2016, who demonstrated that legumes had higher N and lower NDF contents than grasses [21]. Acid detergent fiber (ADF) serves as an approximation for the portion of the forage that animals cannot digest, with ADF representing dry matter digestibility. This parameter pertains to the structural elements of the forage, composed of cellulose and lignin. Higher ADF values correspond to a reduced digestibility of forages by animals. According to previous data [16], a low lignin lucerne monoculture had the lowest ADF, and grass monocultures had the highest ADF; in our study, no significant differences in tendency were found when comparing the perennial ryegrass and ×*Festulolium* monoculture swards with other swards that contained lucerne or other legume grass. A low ADF content in swards is influenced by factors such as plant species, growing conditions, harvest timing, and other environmental and agronomic factors [37]; an earlier harvest may have fewer mature plants with a lower fiber content, some plant species are naturally more nutritious, and some grass varieties can be genetically modified to contain fewer fibers.

Also, our study confirms that monoculture grass sward productivity requires the application of N fertilizers [38]. Meanwhile, several researchers have obtained similar results, as long as the proportion of mixed grass–legume swards is sufficient to take up mineral N from the soil [39], it can be expected that this will prevent N losses via leaching [40]. Dindova et al. (2019) studied the relationships between long-term fertilization management and forage nutritive value in grasslands and also found that the effect of fertilization on the proportion of functional groups was visible across cuts and years [27]. These results are similar to our study results demonstrating increased proportions of grass under increasing N fertilization and consequent reductions in legumes. This can be elucidated by the accelerated growth of the grasses in response to nitrogen fertilization during the spring season. Despite nitrogen application occurring in early spring, distinctions in functional groups among the fertilization treatments were evidently noticeable throughout the cutting periods [27]. It was also confirmed by Meo-Filho et al. (2023) that a grass–clover mix not only reduces fertilizer use but produces silage with less protein [41]. Incorporating forage legumes into crop production systems offers multiple benefits, as they not only serve as a source of food and feed for animals but also enhance soil productivity while serving as soil-conserving elements within agricultural systems [14].

## 4. Materials and Methods

### 4.1. Experimental Site

A field experiment with a mixture of legumes and grasses was conducted from 2018 to 2021 at the Lithuanian Research Centre for Agriculture and Forestry, located in Akademija (55°22′59.7″ N 23°51′42.1″ E), Central Lithuania, in soil formed from loamy clay, classified as Endocalcaric Epigleyic Cambisol (WRB, 2014) [42]. The presented study does not include the season in which the experimental field was set up, i.e., 2018. The content of the available macronutrients, measured before the experiment started in 2018 (in the soil’s arable layer of 0–25 cm) was very high for phosphorus (P) (98 mg kg^−1^) and for potassium (K) (144 mg kg^−1^), and relatively high for organic carbon (C_org_) (1.76%) and total nitrogen (N) (0.247%). The soil pH was in the neutral range (pH 6.9). The content of the available macronutrients, which were measured at the same time, was, in general, sufficient for pasture plants.

### 4.2. Weather Conditions

**Figure 6:**
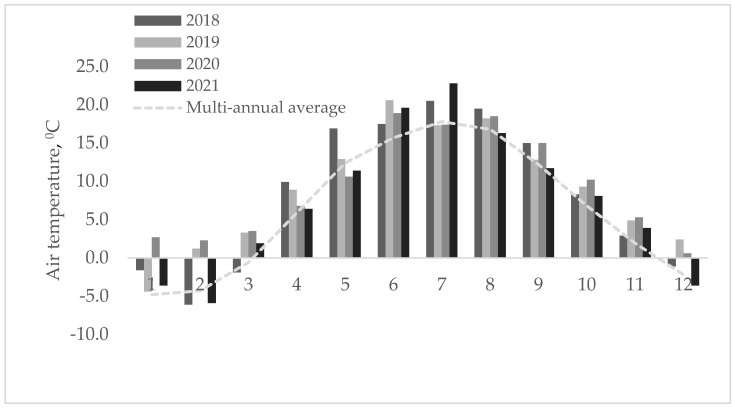

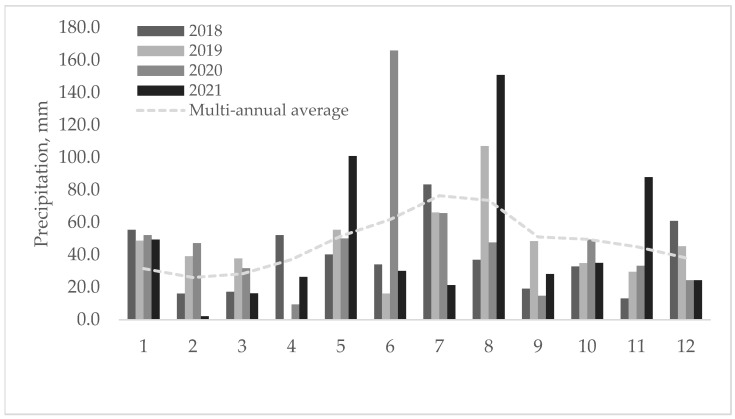
Average monthly air temperature and cumulative precipitation in 2018–2021.

The weather conditions varied during the experiment’s duration. The average monthly air temperature and the cumulative precipitation are shown in Figure 6.

According to the Lithuanian Hydrometeorological Service, since 2021, the new average annual air temperature in Lithuania has remained at 7.4 °C (1991–2020 climate norm), with an average precipitation of 675 millimeters. Compared to the previous norm (1981–2010 climate norm), the average precipitation has decreased by 3 percent, and the average air temperature has increased by 0.4 °C. At the beginning of the experiment, the year of sowing was favorable for plant growth, with high humidity and heat, and there were suitable conditions for plant germination and swelling. The most unfavorable years for plant growth were 2019 and 2021 due to low rainfall and higher-than-usual temperatures. The meteorological data used were from the Dotnuva meteorological station, which is less than three km away from the experimental site.

### 4.3. Experimental Design

The field experiment, arranged in a two-factor split-plot design and replicated four times, begun in spring 2018. Before sowing, the experimental field was fertilized with 5–20.5–36 kg N–P–K ha^−1^. The sowing mixtures, composed of Lithuanian cultivars, namely, white clover (*Trifolium repens* L.) cv. ‘Dotnuviai’, red clover (*Trifolium pratense* L.) cv. ‘Sadūnai’, alfalfa (*Medicago sativa* L.) cv. ‘Malvina’, sainfoin (*Onobrychis viciifolia* Scop.) cv. ‘Meduviai’, perennial ryegrass (*Lolium perenne* L.) cv. ‘Elena DS’, ×*Festulolium* (×*Festulolium* Asch. & Graebn.) cv. ‘Vėtra’, meadow fescue (*Festuca pratensis* Huds.) cv. ‘Raskila’, and timothy (*Phleum pratense* L.) cv. ‘Dubingiai’, were sown on plots of 15 m^2^ (1.5 × 10 m) at an amount of 10, 15, 15, 80, 18, 18, 20, and 12 kg of seed material per 1 ha. The experiment was designed for the multipurpose (mixed) use of swards (Table 6). Factor A: different combinations of plant species; Factor B: mineral nitrogen fertilization (150 kg N ha^−1^ per year) starting with the first year of sward use. This experiment had one field not fertilized.

The swards were fertilized three times per season: at the beginning of the growing season, with 60 kg N ha^−1^, and after the first and second cuts, each time with 45 kg N ha^−1^. To determine the productivity of the sward, the plants were cut according to the dominant plant species in the sward at the beginning of the heading stage of the grasses.

### 4.4. Plant Sampling and Measurements

The number of harvest cuts depended on the growing conditions in the individual years, with 4, 5, and 4 cuts in 2019, 2020, and 2021, respectively. To determine the dry matter yield (DMY), samples of 1–1.5 kg fresh biomass were oven-dried at a 105 °C temperature to a constant weight. Samples used for the determination of the chemical composition and nutritional value of the swards were taken in the same order, and, to avoid changes in the chemical composition of the grass, the samples were dried immediately after cutting at 60 °C for 48 h in a well-ventilated oven. After drying, the samples were ground and prepared for chemical analyses using a perennial grass analysis instrument, a NIR 6500 spectrometer (FOSS, Hilleroed, Denmark). 

The quality indicators of the swards were determined by choosing the most productive first cut, which was intended for winter fodder. For a comparison of the quality of the grasses, the mid-season harvest, the third cut, was also chosen.

### 4.5. Statistical Analysis

A two-factor analysis of variance (ANOVA) with the statistical package STATISTICA 8 was used to process the data to investigate the effect of nitrogen fertilizer on sward quality with different species compositions. Differences between the treatments analyzed were determined using a post hoc exploratory analysis at the 5% level of significance (*p* < 0.05).

## Figures and Tables

**Table 1:** Significance of the chemical composition of aboveground biomass of multi-species swards in 2019–2021.

Year	2019		2020		2021	
Cuts	I	III	I	III	I	III
Factor A (grass–legume mixtures)	*	*	*	*	*	*
Factor B (fertilization)	ns	*	*	*	*	*
Interaction A × B	*	*	*	*	*	ns

* Values with asterisks indicate significant differences (* *p* < 0.05), and ns indicates non-significant difference.

**Table 2:** Significance of the chemical composition of aboveground biomass of multi-species swards in 2019–2021.

Year	2019		2020		2021	
Cuts	I	III	I	III	I	III
Factor A (grass–legume mixtures)	*	*	*	*	*	*
Factor B (fertilization)	*	ns	*	*	*	ns
Interaction A × B	ns	*	ns	*	ns	*

* Values with asterisks indicate significant differences (* *p* < 0.05), and ns indicates non-significant difference.

**Table 3:** Significance of the chemical composition of aboveground biomass of multi-species swards in 2019–2021.

Year	2019		2020		2021	
Cuts	I	III	I	III	I	III
Factor A (grass–legume mixtures)	ns	*	*	*	*	*
Factor B (fertilization)	*	*	*	*	*	ns
Interaction A × B	ns	*	*	*	ns	ns

* Values with asterisks indicate significant differences (* *p* < 0.05), and ns indicates non-significant difference.

**Table 4:** Significance of the chemical composition of aboveground biomass of multi-species swards in 2019–2021.

Year	2019		2020		2021	
Cuts	I	III	I	III	I	III
Factor A (grass–legume mixtures)	*	*	*	*	*	*
Factor B (fertilization)	*	*	*	*	ns	*
Interaction A × B	*	*	*	*	*	*

* Values with asterisks indicate significant differences (* *p* < 0.05), and ns indicates non-significant difference.

**Table 5:** Significance of the chemical composition of aboveground biomass of multi-species swards in 2019–2021.

Year	2019		2020		2021	
Cuts	I	III	I	III	I	III
Factor A (grass–legume mixtures)	ns	*	*	*	*	ns
Factor B (fertilization)	*	*	ns	ns	*	ns
Interaction A × B	ns	ns	*	ns	*	ns

* Values with asterisks indicate significant differences (* *p* < 0.05), and ns indicates non-significant difference.

**Table 6:** The types of swards used in the experiment; the numbers in the columns indicate the targeted proportion of the species at sowing.

Treatment	Number of Grass sp.	Number of Legume sp.	Perennial Ryegrass (G1)	×*Festulolium* (G2)	Meadow Fescue (G3)	Timothy (G4)	White Clover (L1)	Red Clover (L2)	Lucerne (L3)	Sainfoin (L4)
1	1	0	100							
2	1	0		100						
3	1	2	60				20	20		
4	1	2		60			20	20		
5	2	2	30	30			20	20		
6	4	2	15	15	15	15	20	20		
7	2	2	30	30			20		20	
8	4	2	15	15	15	15	20		20	
9	2	2	30	30			20			20
10	4	2	15	15	15	15	20			20
11	2	4	30	30			10	10	10	10
12	4	4	15	15	15	15	10	10	10	10

## Data Availability

Not applicable.
